# Whole-Genome Sequencing of a Salmonella enterica subsp. enterica Serovar Infantis Strain Isolated from Broiler Chicken in Peru

**DOI:** 10.1128/MRA.00826-19

**Published:** 2019-10-24

**Authors:** Katherine Vallejos-Sánchez, Luis Tataje-Lavanda, Doris Villanueva-Pérez, Jorge Bendezú, Ángela Montalván, Mirko Zimic-Peralta, Manolo Fernández-Sánchez, Manolo Fernández-Díaz

**Affiliations:** a Laboratorios de Investigación y Desarrollo, FARVET, Chincha Alta, Ica, Peru; b Laboratorio de Bioinformática, Biología Molecular y Desarrollos Tecnológicos. Laboratorios de Investigación y Desarrollo, Facultad de Ciencias, Universidad Peruana Cayetano Heredia, Lima, Peru; Loyola University Chicago

## Abstract

This report shows the whole-genome sequence of the multidrug-resistant Salmonella enterica subsp. enterica serovar Infantis strain FARPER-219. Antibiotic resistance genes are found mainly in the plasmid. Our findings show important genetic information that provides an understanding of the recent spread of this serotype in poultry.

## ANNOUNCEMENT

Salmonella enterica subsp. enterica serovar Infantis (*S.* Infantis) is an emerging serotype in poultry, reflected by an increased prevalence in poultry flocks, in broiler meat, and in human foodborne illness cases (1–4), producing a significant economic loss for the poultry industry (5). In several countries, this serotype has been reported to be associated with a high prevalence and antimicrobial resistance (6–9).

In Peru, similar data have been reported, showing that *S.* Infantis is the most prevalent serotype (91.43%) in chicken farms (10) and the third most frequently isolated strain from humans and food (11–13). However, there is limited information on full-genome sequences of *S.* Infantis, mainly isolated from poultry.

Here, we report the whole-genome sequence and the annotation of the FARPER-219 strain. It was isolated from a small farm in the southern region of Peru in 2017 from the liver and spleen of broiler chickens. The strain was identified as S. enterica using colony morphology and specific DNA PCR (14). Genomic DNA was extracted from bacterial culture (brain heart infusion [BHI] broth at 37°C overnight) using a phenol-chloroform protocol (15). Genome sequencing was performed with a 20-kb SMRTbell library (PacBio DNA/polymerase binding kit P6) on the PacBio RS II platform (PacBio DNA sequencing kit 4.0) using C4 chemistry with 8 single-molecule real-time (SMRT) cells (Macrogen, Inc., South Korea). Genome assembly was performed *de novo* using Hierarchical Genome Assembly Process (HGAP) version 3.0 (16) from the SMRT portal version 2.3, with default parameters, by Macrogen, Inc.

**FIG 1:**
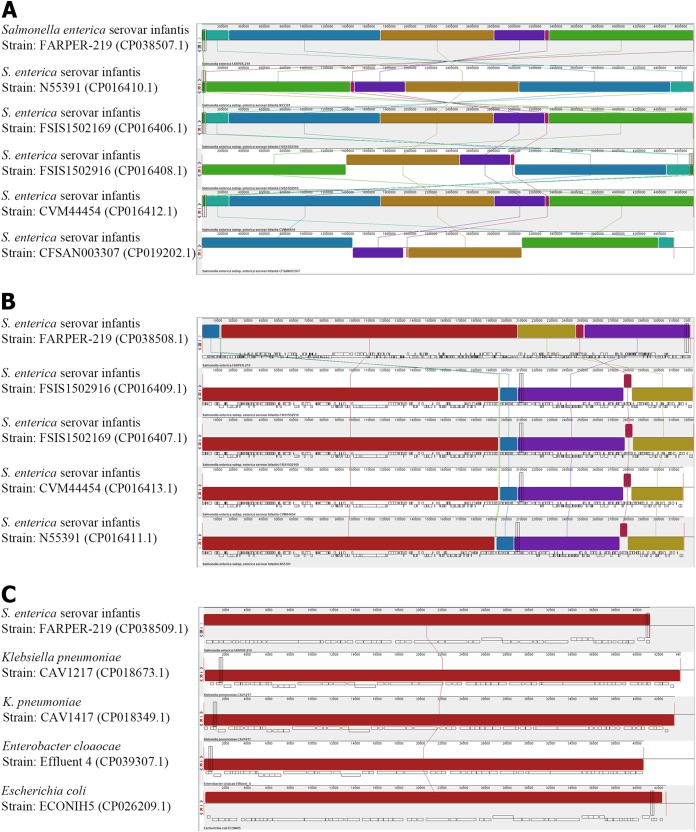
Synteny between the FARPER-219 genome and similar genomes. Pairwise alignments were generated with progressiveMauve. Accession numbers are indicated on the left. Colored boxes indicate synteny regions that aligned to other genomes, and colored lines connect regions. (A) Chromosome; (B) plasmid; and (C) lineal contig.

A total of 99,112 reads (average length, 7,478 bp; *N*_50_, 11,183 bp) were generated, and the assembled genome resulted in one closed circular chromosome of 4,727,696 bp (G+C content of 52.3%; coverage, 105×), including a circular plasmid of 320,892 bp (G+C content of 50.4%; coverage, 99×) and one linear contig of 41,193 bp (G+C content of 34.5%; coverage, 231×). The NCBI Prokaryotic Genome Automatic Annotation Pipeline version 4.7 (17) identified 4,717 genes, 4,598 coding DNA sequences (CDSs), and 119 RNA genes (22 rRNAs, 84 tRNAs, 1 transfer-messenger RNA [tmRNA], and 12 noncoding RNAs [ncRNAs]) on the chromosome; 369 genes, 368 CDSs, and 1 tRNA on the plasmid; and 55 genes and 55 CDSs on the linear contig. In order to corroborate the assemblies, we performed a multiple sequence alignment by Mauve version 20150226 (18) for synteny analysis (Fig. 1).

The multilocus sequence typing (MLST) profile of the genome (sequence type 32 [ST-32]) and the plasmid (IncI1) was performed using MLST version 2.0 (19) and pMLST version 2.0 (20), respectively. Using ResFinder version 3.1 (21), a total of 9 genes associated with antimicrobial resistance were found in the plasmid, namely, *aac(3)-IV*, *aph(4)-Ia*, and *aadA1*, which confer aminoglycoside resistance; *bla*_CTX-M-65_, which confers beta-lactam resistance; *fosA3*, which confers fosfomycin resistance; *floR*, which confers phenicol resistance; *sul1*, which confers sulfonamide resistance; *tet*(A), which confers tetracycline resistance; and *dfrA14*, which confers trimethoprim resistance. Only one antimicrobial resistance gene was found in the chromosome, namely, *aac(6′)-Iaa*, which confers aminoglycoside resistance.

The similarity of the strain FARPER-219 to other strains was measured by BLASTn analysis of the 16S rRNA gene, and genomic average nucleotide identity (ANI) was calculated with ANI Calculator (22). FARPER-219 showed 99.96% ANI and 100% 16S pairwise identity with *S.* Infantis FSIS1502916 (GenBank accession number CP016408), isolated from ground chicken (United States) (23), suggesting a monophyletic lineage. BLASTn analysis of the linear contig indicated 97.66% identity and 92% coverage (nucleotide database) with two extrachromosomal segments of Klebsiella pneumoniae, namely, lineal (GenBank accession number CP018673) and circular (GenBank accession number CP018349) segments.

This study provides important genome information to facilitate molecular studies of *S.* Infantis in the poultry farms and to establish a basis on which to improve epidemiological surveillance of this important serovar. In addition, future studies are needed to clarify the presence of the extrachromosomal segment of Klebsiella pneumoniae in *S.* Infantis, as it had not been previously reported.

### Data availability.

The genome sequence of FARPER-219 has been deposited in the GenBank database under accession numbers CP038507, CP038508, and CP038509. Raw data are available in BioSample accession number SAMN11252736 and SRA run number SRR8903487.
